# Blood pressure signature genes and blood pressure response to thiazide diuretics: results from the PEAR and PEAR-2 studies

**DOI:** 10.1186/s12920-018-0370-x

**Published:** 2018-06-20

**Authors:** Ana Caroline C. Sá, Amy Webb, Yan Gong, Caitrin W. McDonough, Mohamed H. Shahin, Somnath Datta, Taimour Y. Langaee, Stephen T. Turner, Amber L. Beitelshees, Arlene B. Chapman, Eric Boerwinkle, John G. Gums, Steven E. Scherer, Rhonda M. Cooper-DeHoff, Wolfgang Sadee, Julie A. Johnson

**Affiliations:** 1 Center for Pharmacogenomics, Department of Pharmacotherapy and Translational Research, College of Pharmacy, University of Florida, P.O.Box 100484, Gainesville, FL 32610-0486 USA; 2 Graduate Program in Genetics and Genomics, University of Florida, Gainesville, FL USA; 3 Department of Biomedical Informatics, College of Medicine, The Ohio State University, Columbus, OH USA; 4 Department of Biostatistics, University of Florida, Gainesville, FL USA; 5 Division of Nephrology and Hypertension, Mayo Clinic, Rochester, MN USA; 6 Division of Endocrinology, Diabetes and Nutrition, University of Maryland, Baltimore, MD USA; 7 Department of Medicine, University of Chicago, Chicago, IL USA; 8 Division of Epidemiology, University of Texas at Houston, Houston, TX USA; 9 Department of Pharmacotherapy and Translational Research, College of Pharmacy, University of Florida, Gainesville, USA; 10 Department of Community Health and Family Medicine, College of Medicine, University of Florida, Gainesville, FL USA; 11 Human Genome Sequencing Center, Baylor College of Medicine, Houston, TX USA; 12 Department of Medicine, Division of Cardiovascular Medicine, University of Florida, Gainesville, FL USA; 13 Department of Cancer Biology and Genetic, College of Medicine, Center for Pharmacogenomics, Ohio State University, Columbus, OH USA

**Keywords:** Pharmacogenomics, Hypertension, Thiazide diuretics, Personalized medicine, RNA-Seq, eQTL

## Abstract

**Background:**

Recently, 34 genes had been associated with differential expression relative to blood pressure (BP)/ hypertension (HTN). We hypothesize that some of the genes associated with BP/HTN are also associated with BP response to antihypertensive treatment with thiazide diuretics.

**Methods:**

We assessed these 34 genes for association with differential expression to BP response to thiazide diuretics with RNA sequencing in whole blood samples from 150 hypertensive participants from the Pharmacogenomic Evaluation of Antihypertensive Responses (PEAR) and PEAR-2 studies. PEAR white and PEAR-2 white and black participants (*n* = 50 for each group) were selected based on the upper and lower quartile of BP response to hydrochlorothiazide (HCTZ) and to chlorthalidone.

**Results:**

*FOS, DUSP1* and *PPP1R15A* were differentially expressed across all cohorts (meta-analysis *p*-value < 2.0 × 10^− 6^), and responders to HCTZ or chlorthalidone presented up-regulated transcripts. Rs11065987 in chromosome 12, a *trans*-eQTL for expression of *FOS*, *PPP1R15A* and other genes, is also associated with BP response to HCTZ in PEAR whites (SBP: β = − 2.1; *p* = 1.7 × 10^− 3^; DBP: β = − 1.4; *p* = 2.9 × 10^− 3^).

**Conclusions:**

These findings suggest *FOS, DUSP1* and *PPP1R15A* as potential molecular determinants of antihypertensive response to thiazide diuretics.

**Trial registration:**

NCT00246519, NCT01203852 www.clinicaltrials.gov

**Electronic supplementary material:**

The online version of this article (10.1186/s12920-018-0370-x) contains supplementary material, which is available to authorized users.

## Background

Hypertension (HTN) is the most important modifiable risk factor for cardiovascular diseases- coronary artery disease, myocardial infarction, heart failure, stroke and peripheral vascular diseases; controlling blood pressure (BP) is critical for reducing long-term mortality and morbidity rates [1]. Despite the plethora of therapeutic options, selection of the initial anti-HTN treatment remains empirical. Worldwide, 1 billion people suffer from HTN [2] but only about 50% of those under drug therapy achieve the treatment goal, which highlights that anti-HTN drug selection for a specific patient likely impacts therapy success [3, 4].

Thiazide diuretics (TD) are a centerpiece of anti-HTN therapy due to their effectiveness, and safety profile in the management of HTN. Among the available anti-HTN medications, HCTZ, chlorthalidone and other TD are considered first line options for most patients with uncomplicated essential HTN, and are highly recommended for patients requiring more than one anti-HTN therapy for control of BP [5]. However, TD have variable efficacy, and less than 50% of HCTZ-treated patients achieve BP control [3]. The inter-individual variability in BP response to TD is likely to contribute to suboptimal BP control.

Using a genome-wide association (GWAS) approach, two replicated regions, one in PRKCA (protein kinase C, alpha) and the other one near GNAS (G protein alpha subunit), were identified with clinically relevant effects on BP response to HCTZ [6]. Despite the successes, the GWAS approach provides only one dimension of molecular information about BP response to anti-HTN treatment. While it is a critical dimension, analyzing DNA variation alone is insufficient for achieving an understanding of the multidimensional complexity of BP response to TD. In this context, transcriptomics (gene expression profiling) has been described as an innovative approach that enables biomarker discovery associated with different diseases and traits [7–10].

Recently, Huan et al. [9] identified 34 genes associated with differential expression relative to BP/HTN, which in aggregate explain ~ 9% of inter-individual variability in BP. In addition, previous findings suggest that some signals from HTN GWAS may predict anti-HTN drug response [11]. This study tests the hypothesis that some of the differentially expressed genes associated with BP/HTN are also associated with BP response to antihypertensive treatment with TD. We assessed the association of these 34 genes with differential expression to BP response to TD by applying RNA sequencing data from the Pharmacogenomic Evaluation of Antihypertensive Responses (PEAR) and PEAR-2 studies.

## Methods

### Study population and ethics statement

This study includes data from PEAR and PEAR-2 (NCT00246519, NCT01203852 www.clinicaltrials.gov), which were previously described in details [12]. Briefly, PEAR was a multicenter, randomized clinical trial with the primary aim of evaluating the role of genetic variability on BP response of HCTZ and/or atenolol treated patients. Study participants (*n* = 768) with uncomplicated HTN were randomized to receive monotherapy of either the thiazide diuretic HCTZ, or the beta-blocker atenolol for a period of 9 weeks. Fasting blood and urine samples were collected at baseline (untreated), after 9 weeks of monotherapy, and after 9 weeks of combination therapy. BP responses were assessed using office, home, and 24-h ambulatory BP and then a composite BP response was constructed [13].

The PEAR-2 clinical trial included a hypertensive population similar to the one in PEAR, and for which metoprolol, a beta-blocker, and chlorthalidone, a thiazide-like diuretic, were tested. Details of this prospective, clinical trial were previously published [14]. Briefly, 417 hypertensive participants were treated in a sequential monotherapy design with metoprolol and then chlorthalidone with at least 4 week washout periods prior to each active treatment. Data collected included home and clinic BP measurements, adverse metabolic effects, fasting whole blood, and urine samples.

### Gene expression profile with RNA-Seq

PEAR whites and PEAR-2 white and black participants were selected for gene expression profiling with RNA-Seq based on the differences in their BP response to HCTZ and chlorthalidone treatment, respectively. A total of 150 patients with BP responses to either HCTZ or chlorthalidone in the top and bottom quartiles from each of the three cohorts were selected and classified as poor BP responders (non-responders) and good BP responders (responders). Sample size was selected based on the theoretical statistical calculations [15], which revealed that with 32 million reads per sample (average number of RNA-Seq reads generated), coefficient of variation (σ) = 0.8, two-sided α level = 0.05, and 25 samples per group (25 responders and 25 non-responders to thiazide diuretics) we have greater than 80% power to detect two-fold differences in expression.

We determined the mean changes of serum potassium concentrations and uric acid levels in non-responders before and after treatment with HCTZ and chlorthalidone with the premise that if the cause of the nonresponse for BP lowering was nonadherence, then these individuals would also not have any adverse metabolic responses that are typically seen with thiazide. We also compared changes from baseline to after treatment serum potassium and uric acid using paired t-tests. Potassium depletion and uric acid elevation are commonly observed secondary to treatment with TD [16–18], and were lab parameters with statistically significant change in the overall clinical study from PEAR participants [19, 20].

Using whole blood samples collected before HCTZ or chlorthalidone monotherapy, RNA was extracted using the PAXgene Blood RNA kit IVD (Qiagen, Valenica, CA). The selection of poly(A) mRNA from total RNA was performed using Sera-Mag Magnetic Oligo(dT) Beads (Illumina, San Diego,CA) according to the manufacturer’s protocol. 100 ng of RNA was then used as a template for cDNA synthesis. Libraries were prepared following the strand-specific protocol [21]. DNA clusters were generated using the Illumina cluster station, followed by 100 cycles of paired-end sequencing on the Illumina HiSeq 2000, performed at Baylor Human Genome Sequencing Center in Texas. For data quality control purposes, read duplicates removal was implemented using Picard (http://broadinstitute.github.io/picard/) MarkDuplicates option.

The 100 bp reads generated in the paired-end RNA sequencing were uniquely mapped to the human reference genome (hg19) using TopHat v2.0.10 [22] allowing for four reads mismatches, read edit distance of six, one mismatch in the anchor region of a spliced read, and a maximum of five multi-hits. Transcript assembly was performed using Cufflinks v2.2.1. Statistical analysis were carried out with Cuffdiff and gene expression levels are reported in fragments per kilobase per million reads (FPKM), considering reads mapped to exonic regions of the 34 genes previously associated with BP/HTN [9].

Additionally, we performed differential expression analysis using alternative tools in order to adjust the expression levels for age, sex and baseline diastolic BP because we observed, for these variables, statistically significant differences between participants classified as responders and non-responders to thiazide diuretics (Table 1). Other common covariates in association studies of hypertension, such as Body Mass Index (BMI) and smoking were not included as covariates because previous analysis of BP response in PEAR [23] established that these variables were not associated with BP response. By using BAM files from TopHat 2 alignments, we were able to count the number of reads for each known human genes (Gencode gene annotation release 18) applying the htseq-count function from the HTSeq bioconductor package [24]. Counts were modeled to a Negative Binomial distribution using a generalized linear model in edgeR [25].

**Table 1:** Characteristics of PEAR and PEAR-2 participants classified as responder and non-responders for the RNA-Seq analysis

	Whites (*n* = 99)				Blacks (*n* = 50)	
	HCTZ		Chlorthalidone		Chlorthalidone	
Characteristics	Responders (*n* = 24)	Non-responders (*n* = 25)	Responders (*n* = 25)	Non-responders (*n* = 25)	Responders (*n* = 25)	Non-responders (*n* = 25)
Age	48 ± 12	48 ± 8	53 ± 8	48 ± 10	52 ± 8	50 ± 10
Female, n (%)	11 (44%)	10 (40%)	15 (75%)^a^	5 (25%)^a^	12 (48%)	12 (48%)
BMI, kg^a^m^− 2^	29 ± 5	32 ± 6	32 ± 5	30.5 ± 5	30 ± 6	31 ± 5
Baseline DBP	93 ± 5	94 ± 4	97 ± 6^a^	93 ± 5^a^	98 ± 6^a^	93 ± 4^a^
Baseline SBP	146 ± 10	144 ± 10	152 ± 11^a^	144 ± 9^a^	152 ± 10^a^	146 ± 10^a^
DBP response to TD	−9 ± 6^b^	0.06 ± 4^b^	− 14 ± 4^b^	− 0.2 ± 2^b^	− 17 ± 4^b^	− 1.4 ± 3^b^
SBP response to TD	−12 ± 6^b^	− 0.9 ± 6^b^	− 22 ± 7^b^	− 1.5 ± 5^b^	− 27 ± 7^b^	− 4.4 ± 5^b^

Mean and Standard Deviation values for the continuous variables were presented

*BMI* body mass index, *SBP* systolic blood pressure, *DBP* diastolic blood pressure, *TD* thiazide diuretics

^a^Significant at the 0.05 probability level

^b^Significant at the 0.001 probability level

### Statistical methods

Based on the fact that the BP signature genes, selected for this analysis, were discovered in whites, the primary data analysis was also performed in whites treated with HCTZ or chlorthalidone. Associations of differences in expression levels of these genes in responders compared to non-responders to TD was evaluated using a t-test to quantify the statistical significance in the differences observed among the gene expression measurements (FPKM). Bonferroni corrected *P* values < 0.0015 (0.05/34) were considered statistically significant. In addition, we assessed the statistical significance (hypergeometric test) of the overlap between the 34 BP signature genes and the 29 genes associated with thiazide diuretics blood pressure response at the whole transcriptome level (FDR *p*-value < 0.05) [29].

For each differentially expressed gene in PEAR or in PEAR-2 whites (6 in total), we attempted replication in PEAR-2 blacks and the alternate group of whites in order to validate the association of the genes with BP response to TD. A strict approach was established for validation with Bonferroni corrected *P* value (< 0.05/6 = 0.008) and the same fold change direction (either up or down regulation) as the primary analysis in whites treated with HCTZ or chlorthalidone.

For those genes that passed the validation criteria, the differential expression results from each study cohort were combined in a meta-analysis, using standardized *p*-values to follow the assumption of the Fisher p-value combination method implemented by the R package MetaRNASeq [26]. We considered that genes with meta-analysis *p* values < 2.0 × 10^− 6^ (0.05/25,000) achieved transcriptome-wide association with BP response to TD.

To evaluate whether *FOS, DUSP1* and *PPP1R15A* robustly predict BP response to TD, PEAR participants were assigned into the derivation cohort for logistic regression model building. PEAR-2 whites constituted the validation cohort, in which area under the receiver operator curve was calculated in the R ROCR package [27], for model evaluation. The TD prediction model was compared to logistic regression model including randomly selected genes from whole transcriptome analysis. Twenty randomly selected genes were sampled (R function “sample”, 20 rounds), and each random signature was fitted to a logistic regression model to assess the probability of random gene signature performing better than the TD genes. Gene expression measures in FPKM were used for this analysis.

### Genomics analysis

Previous studies have explored the genome-wide genotyping results for the PEAR and PEAR-2 studies in much more detail [6, 11]. GWAS data for chlorthalidone in PEAR-2 will be reported separately. Briefly, DNA samples were genotyped using Illumina Human Omni-1Million Quad BeadChip and 2.5 M-8 BeadChip (Illumina, San Diego CA) for PEAR and PEAR-2, respectively. Genotypes were called using GenTrain2 clustering algorithm (GenomeStudio, Illumina, San Diego CA). MaCH software (version 1.0.16) was used to impute SNPs based on HapMapIII haplotypes.

In order to identify SNPs potentially regulating the expression of the genes differentially expressed in the RNA-Seq data, we consulted the Blood eQTL browser [28]. The SNPs identified as eQTL for the differentially expressed genes were then evaluated in the PEAR and PEAR2 GWAS data, to test for a genetic association with BP response to TD. SNP associations with BP response were evaluated using previously conducted GWAS analyses [6] that included data on systolic and diastolic BP responses to HCTZ in 228 whites participants from PEAR, and responses to chlorthalidone in 185 white and 142 black participants from PEAR-2. PLINK software was used to run the analysis with adjustment for age, sex, pre-HCTZ/chlorthalidone BP and population substructure by considering the first and second principal components (PC1 and PC2) in all our analysis.

## Results

Table 1 summarizes baseline and demographic characteristics from PEAR white participants treated with HCTZ and PEAR-2 white and black participants treated with chlorthalidone who were selected for RNA-Sequencing. For PEAR, age, body mass index (BMI), sex and baseline BP were not statistically different between participants classified as responders and non-responders to HCTZ. However, in PEAR-2 white participants, differences in sex and baseline BP were statistically significant between responders and non-responders to chlorthalidone. Differences in baseline BP were also observed in PEAR-2 blacks between responders and non-responders to chlorthalidone.

After treatment with HCTZ and chlorthalidone, there were significant reductions on serum potassium concentrations and significant increases serum uric acid levels in participants classified as non-responders (Additional file 1: Table S1). These changes are consistent with previously reported metabolic effects after treatment with TD [19, 20], and suggest high treatment adherence in the group of BP non-responders to TD.

In order to identify genes with differential expression involved in BP response to TD, whole transcriptome sequences were generated from 149 participants treated with HCTZ or chlorthalidone. One of the samples from HCTZ responders did not achieve enough library yield for adequate performance in sequencing. On average, 32 million reads per sample were mapped to the human reference genome (hg 19) and about 93% were uniquely mapped (Additional file 1: Figure S1). Whole transcriptome analyses from the PEAR and PEAR2 studies were previously published [29].

At a Bonferroni corrected alpha (0.0015), six genes were differentially expressed in whites treated with HCTZ or chlorthalidone (Additional file 1: Table S2). Of those *GPR56*, *FOS* and *FGFBP2* were common between the gene lists as significant for BP response to TD at the whole transcriptome level (FDR *p*-value < 0.05 in PEAR or PEAR-2 whites) [29] and the BP signature genes (Additional file 1: Table S2). A hypergeometric test (*p* = 9.0 × 10^− 7^) showed that this overlap between the 29 genes associated to TD BP response at the whole transcriptome level [29] and the 34 BP signature genes is statistically significant. For each gene differentially expressed (*P* < 0.0015) in PEAR or PEAR-2 white participants, we attempted replication in the other group of white study participants and in black participants from PEAR-2 (Additional file 1: Table S2). Of the six genes identified, *FOS* and *DUSP1* were differentially expressed and showed consistent fold change direction in all 3 cohorts (Table 2), passing the stringent Bonferroni corrected alpha at 0.008 for validation. *PPP1R15A* showed consistent directional fold change in all three cohorts, and met the Bonferroni threshold *p* value in PEAR whites given HCTZ (Fold Change (Responders/non-responders): 1.27, *p* = 1.15 × 10^− 3^) and PEAR-2 blacks given chlorthalidone (Fold Change: 1.29, *p* = 1.75 × 10^− 3^), while only achieving nominal significance in PEAR-2 whites (Fold Change: 1.19, *p* = 3.61 × 10^− 2^) (Table 2). The meta-analysis of all participants with RNA-Seq data included *FOS, DUSP1* and *PPP1R15A*, and confirmed transcriptome-wide associations that far exceeded transcriptome wide (and genome wide) significance for *FOS* (*p* = 2 × 10^− 12^)*, DUSP1* (*p* = 9.5 × 10^− 12^) and *PPP1R15A* (*p* = 3.6 × 10^− 8^) expression and BP response to TD (Table 2). Even though the statistical strength of the association lessened after the adjustment for age, sex and baseline BP, the fold change direction remains consistent across PEAR whites and PEAR-2 whites and blacks regardless of the statistical methods used (Additional file 1: Table S3).

**Table 2:** Genes differentially expressed between responders and non-responders to HCTZ and chlorthalidone in all 3 cohorts, with consistent direction and transcriptome-wide statistical significance when meta-analyzed

	HCTZ Whites				Chlorthalidone Whites				Chlorthalidone Blacks				Meta-analysis
Genes	Non-resp.	Resp.	Fold Change	*P* value	Non-resp.	Resp.	Fold Change	*P* value	Non-resp.	Resp.	Fold Change	*P* value	*P* value
FOS	39.2	49.5	1.26	2.90E-03	29.4	38.0	1.29	1.15E-03	24.6	35.9	1.46	5.00E-05	2.08E-12
DUSP1	76.0	105.2	1.38	1.50E-04	71.5	92.8	1.30	1.35E-03	63.3	81.7	1.29	3.55E-03	9.50E-12
PPP1R15A	38.3	48.7	1.27	1.15E-03	29.9	35.5	1.19	3.61E-02	27.6	35.6	1.29	1.75E-03	3.64E-08

Fold change corresponds to gene expression levels in responders divided by levels in non-responders, in fragments per kilobase per million reads (FPKM)

**Fig. 1:**
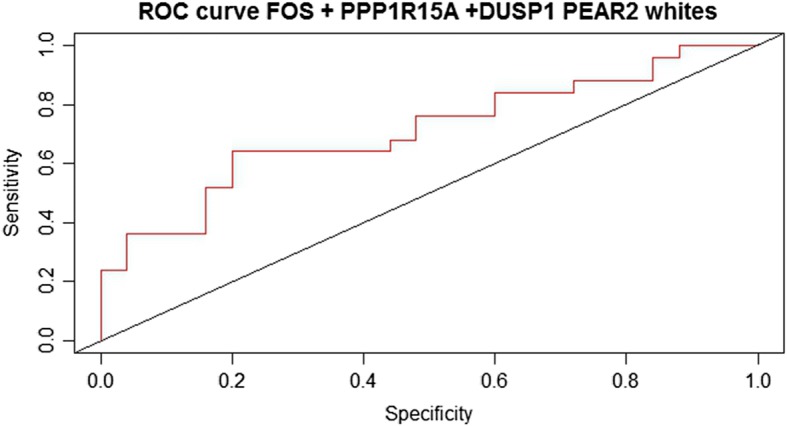
Receiver operator curve for assessment of logistic regression model prediction in PEAR-2. Statistical model including thiazide diuretic (TD) genes FOS, DUSP1 and PPP1R15A showed area under the curve was 0.71, indicative of the model’s good prediction for blood pressure response to TD. Gene expression measures reported in Fragments per Kilobase of Exon per Million mapped (FPKM)

The combination of *FOS, DUSP1* or *PPP1R15A* gene expression in a logistic regression model was statistically significant (*P* = 0.02), and explained 23.3% of the variability in drug response to TD in the derivation cohort (PEAR). For independent assessment of this model in PEAR-2, the area under the curve was 0.71, indicative of the model’s good prediction for BP response to TD (Fig. 1). Additionally, this model performed better than randomly selected signature genes of identical size, re-sampled 20 times (P range: = 0.045–0.96) (Additional file 1: Figure S2).

**Fig. 2:**
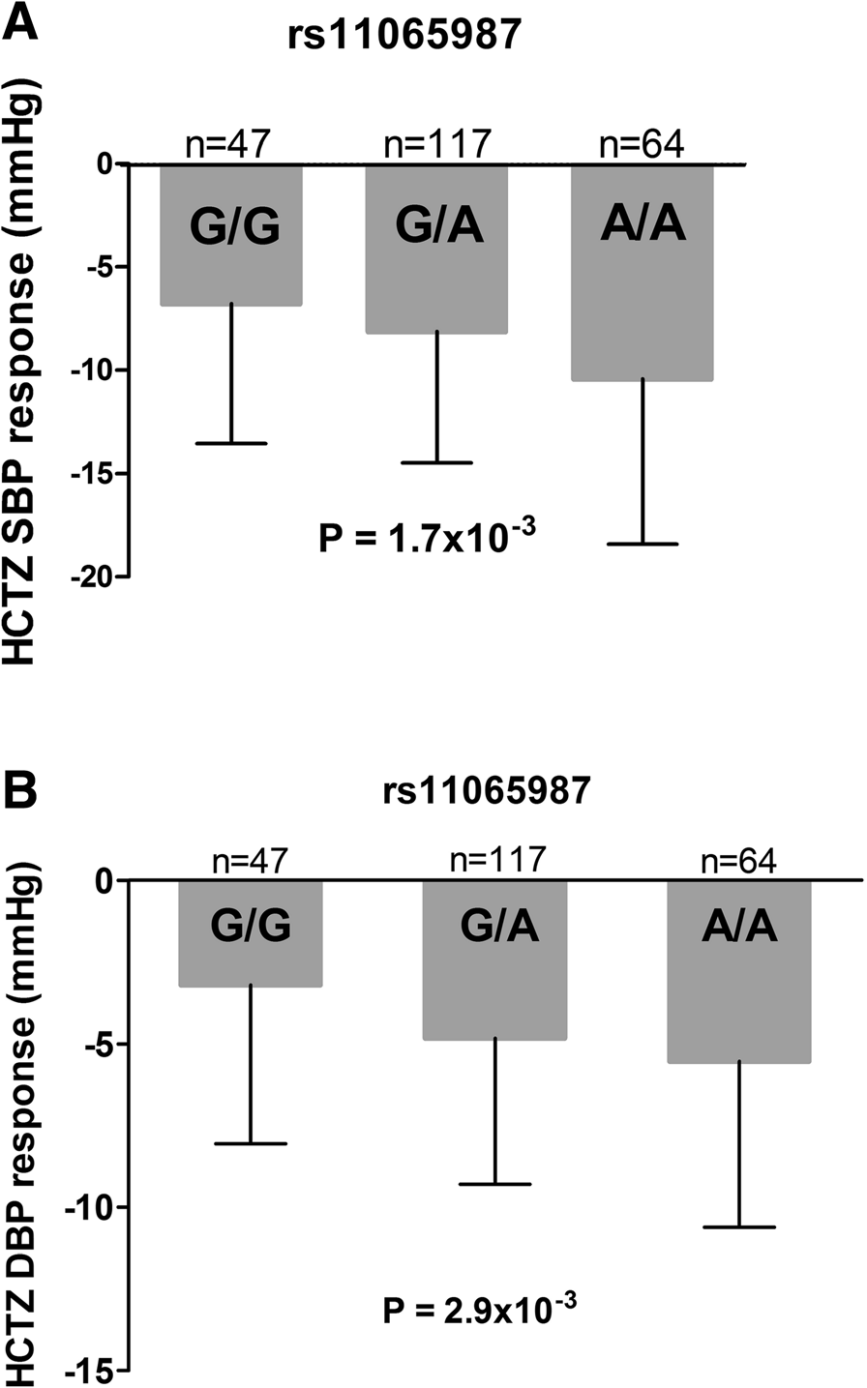
The effect of rs11065987 polymorphism on the blood pressure response of Whites treated with HCTZ in PEAR. Blood pressure responses were adjusted for baseline blood pressure, age, sex, and population substructure. *P*-values represent the contrast of adjusted means between different genotype groups in the PEAR white participants. Error bars represent standard error of the mean. **a** systolic blood pressure response to HCTZ in PEAR whites. **b** diastolic blood pressure response to HCTZ in PEAR whites

Based on data in the Blood eQTL browser [28], we identified 4 trans-eQTLs (rs11065987, rs653178, rs10774625 and rs11066301) associated with reduced expression of both *FOS* and *PPP1R15A* (Additional file 1: Table S4). Because of the high linkage disequilibrium between these SNPs (Additional file 1: Figure S3), we selected a representative SNP (rs11065987) to test for an association with BP response with TD. Rs11065987 was associated with SBP and DBP response to HCTZ in PEAR whites (SBP: β = − 2.1; *p* = 1.7 × 10^–3;^ DBP: β = − 1.4; *p* = 2.9 × 10^− 3^) (Fig. 2) and showed consistent directional association in PEAR-2 whites that did not reach statistical significance in PEAR-2 whites or blacks treated with chlorthalidone (Additional file 1: Table S4).

In order to investigate *FOS* and *PPP1R15A* co-expression, we calculated the Pearson correlation coefficient between the log transformation of expression levels of these genes. *FOS* and *PPP1R15A* showed strong positive correlation with r^2^ = 0.9 in PEAR whites treated with HCTZ and r^2^ = 0.8 in PEAR-2 whites treated with chlorthalidone. This indicates potential common co-regulatory mechanism involving the expression of these genes and driven by rs11065987 or its proxy SNPs.

## Discussion

Despite the widespread use of TD, there is large inter-individual variability in BP or drug response, which has motivated the identification of genetic markers with the potential to optimize antihypertensive treatment selection. GWAS results have definitely contributed to enlarge the current knowledge on the potential role of genetics in inter-individual variability in drug response in general and also to thiazide BP response [6, 11]. However, this approach provides only one dimension of molecular information in thiazide BP response, which may not be sufficient to understand the complexity of this phenotype. Gene expression has been shown to have predictive and prognostic value in disease genomics studies, applying transcriptomics tools for the characterization of novel cancer subtypes [30–32] or the identification of signature genes for hypertension [9, 33], heart failure [34–36] and other diseases. Recent studies presented a remarkable effect of whole transcriptome gene expression data in enriching for drug responders to docetaxel and cisplatin treatment of breast cancer [37], and to erlotinib in non-small cell lung cancer [38]. Each of these studies highlights the potential scientific insights that can be gained through experimental approaches that apply gene expression data. In this study, we investigated differences in gene expression underlying extreme BP response to thiazides in white and black participants from PEAR and PEAR-2. Such approaches have the potential to provide methods for precision medicine, but additionally may provide previously unrecognized insights into BP regulation and responses to antihypertensive drugs.

Herein, we have shown that applying transcriptome sequencing data helped us to identify molecular markers potentially implicated in BP response to TD. Among the 34 genes previously documented to influence BP/HTN, *FOS, DUSP1* and *PPP1R15A* mRNAs were differentially expressed between responders and non-responders in three different cohorts treated with TD, with consistent directional fold change in whites treated with HCTZ and whites and blacks treated with chlorthalidone.

Among these three genes, only *FOS* has been associated previously with the pathophysiology of HTN. Expression of FOS (FBJ murine osteosarcoma viral oncogene homolog, also known as AP-1 transcription factor subunit), a leucine zipper protein that when dimerized with JUN forms a transcription factor complex, is linked to neuronal activation of vasomotor areas in mice [39]. Also, the blockade of *FOS* expression with oligonucleotides attenuates high BP in HTN-induced and spontaneously HTN mice [40].

We did not find any direct evidence in the literature of the involvement of DUSP1 and PPP1R15A that could account mechanistically for a potential susceptibility for HTN and/or BP response to thiazides. However, we found that these genes are involved in biological processes related to BP regulatory mechanisms. For instance, DUSP1 has shown consistent inhibition of ERK 1/2 (Extracellular Regulated Kinases) signaling in vitro and in vivo [41], with potential attenuation on the effects of angiotensin II-mediated vascular smooth muscle cell (VSMC) proliferation and vasoconstriction [42].

PPP1R15A is a regulatory subunit for phosphatase protein (PP) 1 [43]. PP1 is the catalytic subunit for myosin phosphatases, a key convergence point on contractility pathways in VSMC, that dephosphorylates the myosin light chain and initiates the relaxation process for vasodilation [44]. Of relevance, PP1 has a highly specific inhibitor 1 (I-1) which, when activated by protein kinase A, forms a heterotrimeric complex with PP1 and PPP1R15A [43]. This specific interaction of PPP1R15A with the C-terminal region of I-1 engenders strong PP1 inhibition [43] and a potential amplification of contractile response in VSMC [45]. In addition, recent research shows that I-1 regulates thiazide-sensitive NaCl cotransporter (NCC) activity and phosphorylation in the distal convoluted tube (DCT), and loss of I-1 expression in mice lowers arterial BP [46]. Since there is no concrete evidence of the consequences of I-1/PPP1R15A interaction in the regulation of contractile signaling, in VSMC, or in the regulation of NCC activity in DCT, we can only speculate that PPP1R15A may be important for BP regulatory mechanisms. Further experimental validation will be crucial to close the link between PPP1R15A interactions with I-1 for the regulation of PP1 and NCC activity in VSMC and DCT.

In addition, we found rs11065987 associated with both systolic and diastolic BP responses to HCTZ in PEAR whites, and it is also associated in *trans* with decreased expression of two genes in our top list of BP signature genes: *FOS* and *PPP1R15A,* which are co-expressed in the whole blood samples tested in this study. rs11065987, the leading SNP in a small haplotype block, is an intergenic SNP in chromosome 12, where the closest gene is BRCA1 associated protein and previous cardiovascular disease GWA studies identified 12q4 as a risk locus for coronary artery disease and HTN [47]. Further experiments will be valuable to understand the mechanisms involved in gene expression regulation in the chromosome 12q4 region that could potentially affect BP regulation as well.

Although it is not clear how FOS, DUSP1 and PPP1R15A are involved in BP regulation, the differences in gene expression documented in this study taken together with evidence of gene expression regulatory mechanism with *trans*-eQTLs associated with BP response to HCTZ suggest that these genes may be markers of response to TD. Further functional studies may provide additional insights to the field.

This study presents some limitations. First, the number of samples with RNA-Seq data may have limited the power to identify additional genes differentially expressed as well as to validate some of the transcriptomics signals; however, we enhanced the power of the number of samples tested by taking an extreme phenotype approach. Second, using RNA from whole blood for RNA-Seq data analysis may have limited the detection of the expression of some genes/regulatory mechanisms that might be cell type-specific. However, it may be challenging to select only one tissue in order to investigate gene expression as a marker of BP regulation since drug response to anti-HTN might arise from a variety of target tissues such as heart, brain, kidney or vasculature. Not only are these tissues difficult to access in relatively healthy patients, as hypertensive patients are, but it is also not obvious which tissue should be used. Thus we are using whole blood as a surrogate for multiple tissues. Moreover, the original study that served as the basis for selection of BP signature genes also used whole blood samples for that transcriptome-wide gene expression study due to the convenience to identify biomarkers using easily accessible body fluids [9].

## Conclusions

For over half century, thiazide diuretics have been a centerpiece of antihypertensive therapy with more than 100 million prescriptions annually in the US alone. Its large inter-individual variability in BP response emphasizes the need for molecular predictors of drug response that hold potential for improving the antihypertensive therapy. Results of the present study suggest that whole transcriptome data can provide insights into genes potentially involved in the pharmacogenetic phenotype of antihypertensive drug response. We were able to demonstrate that genes previously identified through BP/HTN transcriptome profiling that are also relevant determinants of BP response to TD. Specifically, *FOS, DUSP1* and *PPP1R15A*, through their differential expression, may be involved in the response to TD. To strengthen the finding, through use of a publicly available eQTL database, we found an eQTL (SNP) of *FOS* and *PPP1R15A* that associated with BP response to TD and other SNPs with evidence of gene expression regulatory mechanisms. Further work is needed to understand the mechanistic basis by which differential expression of *FOS*, *DUSP1* and *PPP1R15A* may influence BP regulation and response to TD.

## Additional file

Additional file 1: **Figure S1.** Mapping statistics for PEAR and PEAR-2 RNA-Seq data. **Table S1.** Potassium, glucose and uric acid mean changes in participants classified as non-responders after treatment with HCTZ and chlorthalidone. **Table S2.** Genes previously associated with BP/HTN (34 BP signature genes) and the expression measurements in PEAR and PEAR-2. **Table S3.** Differences in baseline expression levels for *FOS, DUSP1* and *PPP1R15A* between thiazide diuretics responders and non-responders in PEAR and PEAR-2 with adjustment for age, gender and baseline blood pressure. **Figure S2.** *P*-value distribution for association with thiazide diuretics blood pressure response for 20 randomly selected genes. **Table S4.** Representative *trans* eQTL for top differentially expressed genes. **Figure S3.** Linkage disequilibrium plots between rs10655987, rs653178, rs10774625 and rs11066301. (DOCX 5615 kb)

## Abbreviation

BP: Blood Pressure
DBP: Diastolic Blood Pressure
eQTL: Expression Quantitative Trait Loci
ERK: Extracellular Regulated Kinases
FDR: False Discovery Rate
GWAS: Genome-wide Association Studies
HCTZ: Hydrochlorothiazide
HTN: Hypertension
PEAR: Pharmacogenomics Evaluation of Antihypertensives Response
PP: Phosphatase Protein
RNA-Seq: RNA Sequencing
SBP: Systolic Blood Pressure
SNP: Single Nucleotide Polymorphis
VSMC: Vascular Smooth Muscle Cell
