# Supplementary material for: Blood pressure signature genes and blood pressure response to thiazide diuretics: results from the PEAR and PEAR-2 studies
Source: BMC Med Genomics. 2018 Jun 20;11:55. doi: 10.1186/s12920-018-0370-x (PMC6011347; doi:10.1186/s12920-018-0370-x)
Supplement: Supplementary file 1 — Figure S1. Mapping statistics for PEAR and PEAR-2 RNA-Seq data. Table S1. Potassium, glucose and uric acid mean changes in participants classified as non-responders after treatment with HCTZ and chlorthalidone. Table S2. Genes previously associated with BP/HTN (34 BP signature genes) and the expression measurements in PEAR and PEAR-2. Table S3. Differences in baseline expression levels for FOS, DUSP1 and PPP1R15A between thiazide diuretics responders and non-responders in PEAR and PEAR-2 with adjustment for age, gender and baseline blood pressure. Figure S2. P-value distribution for association with thiazide diuretics blood pressure response for 20 randomly selected genes. Table S4. Representative trans eQTL for top differentially expressed genes. Figure S3. Linkage disequilibrium plots between rs10655987, rs653178, rs10774625 and rs11066301. (DOCX 5615 kb) [file 12920_2018_370_MOESM1_ESM.docx]

**Additional file 1**

**Blood Pressure Signature Genes and Blood Pressure Response to Thiazide Diuretics: Results from the PEAR and PEAR-2 Studies**

Ana Caroline C. Sá^1,2^, M.S., Amy Webb^3^, PhD., Yan Gong^1^, PhD., Caitrin W. McDonough^1^, PhD., Mohamed H. Shahin^1^, M.S., PhD., Somnath Datta^4^, PhD., Taimour Y. Langaee^1^, PhD., Stephen T. Turner^5^, M.D., Amber L. Beitelshees^6^, PhD., Arlene B. Chapman^7^, M.D., Eric Boerwinkle^8^, PhD., John G. Gums^9^, Pharm.D., Steven E. Scherer^10^, PhD., Rhonda M. Cooper-DeHoff^1,11^, Pharm.D., M.S., Wolfgang Sadee^12^, PhD., Julie A. Johnson^1, 2,11^, Pharm.D.

1Center for Pharmacogenomics and Department of Pharmacotherapy and Translational Research, University of Florida, Gainesville, FL, USA

2Graduate Program in Genetics and Genomics, University of Florida, Gainesville, FL, USA

3Department of Biomedical Informatics, College of Medicine, The Ohio State University, Columbus, OH, USA

4Department of Biostatistics, University of Florida, Gainesville, FL, USA

5Division of Nephrology and Hypertension, Mayo Clinic, Rochester, MN, USA

6Division of Endocrinology, Diabetes and Nutrition, University of Maryland, Baltimore, MD, USA

7 Department of Medicine, University of Chicago, Chicago, IL, USA

8Division of Epidemiology, University of Texas at Houston, Houston, TX, USA

9Department of Pharmacotherapy and Translational Research, University of Florida, College of Pharmacy and Department of Community Health and Family Medicine, University of Florida College of Medicine, Gainesville, FL, USA

10Human Genome Sequencing Center, Baylor College of Medicine, Houston, TX, USA.

11Division of Cardiovascular Medicine, Department of Medicine, University of Florida, Gainesville, FL, USA

12Center for Pharmacogenomics, Department of Cancer Biology and Genetic, College of Medicine, Ohio State University, Columbus, OH, USA

Corresponding author:

Julie A. Johnson, Pharm.D.

Center for Pharmacogenomics, Department of Pharmacotherapy and Translational Research, College of Pharmacy, University of Florida

P.O.Box 100484 Gainesville, FL 32610-0486

352-273-6309(phone)/352-273-6306 (fax)

Email: johnson@cop.ufl.edu


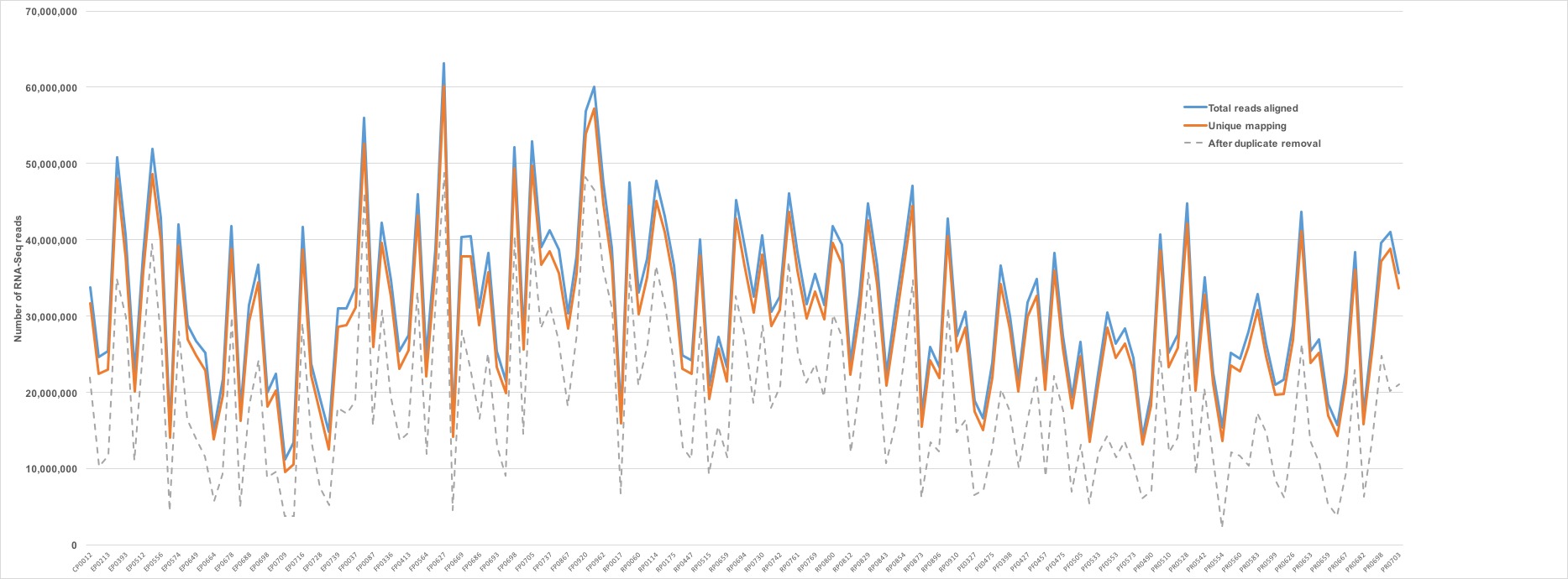


**Figure S1.** Mapping statistics for PEAR and PEAR-2 RNA-Seq data. The blue line represents total number of reads aligned to the human reference genome (hg19) for the 149 samples included in this study. The orange line represents uniquely mapped reads per sample and the dashed line represents total number of reads that remained after duplicate removal with Picard MarkDuplicates option.

**Supplementary Table 1**. Potassium, glucose and uric acid mean changes in participants classified as non-responders after treatment with HCTZ and chlorthalidone.

|  | **Whites** | | | |  | **Blacks** | |
| --- | --- | --- | --- | --- | --- | --- | --- |
|  | **Non-responders to HCTZ (n= 25)** | | **Non-responders to Chlorthalidone (n =25)** | |  | **Non-responders to Chlorthalidone (n=25)** | |
| **Parameters** | **Mean Change** | **P value** | **Mean Change** | **P value** |  | **Mean Change** | **P value** |
| Serum K^+^ (mEq/L) | -0.2±0.4 | 0.016 | -0.6±0.4 | 2.0E-07 |  | -0.45±0.6 | 0.001 |
| Serum glucose, mg/dl | 2.7±12.8 | 0.325 | 6.1±15.3 | 0.059 |  | 2.1±13.0 | 0.445 |
| Serum uric acid, mg/dl | 0.9±1.0 | 9.6E-05 | 1.1±1.0 | 2.8E-05 |  | 1.1±1.4 | 5.6E-04 |
| P values represent the comparison between baseline and the end of the monotherapy | | | | | | | |

**Table S2.** Genes previously associated with BP/HTN^11^ (34 BP signature genes) and the expression measurements in PEAR withes and PEAR-2 whites and blacks treated with HCTZ and chlorthalidone, respectively. Q represents FDR adjusted p-values from whole transcriptome analyses^2^.

| **Gene** | **Chr.** | | **HCTZ WHITES** | | | | | | | | | | **CHLORTHALIDONE WHITES** | | | | | | | | | **CHLORTHALIDONE BLACKS** | | | | | | | |  |
| --- | --- | --- | --- | --- | --- | --- | --- | --- | --- | --- | --- | --- | --- | --- | --- | --- | --- | --- | --- | --- | --- | --- | --- | --- | --- | --- | --- | --- | --- | --- |
|  |  |  | **Fold Change** | | | ***P*** | | | ***Q*** | | | **Fold Change** | | | | ***P*** | | | ***Q*** | | | **Fold Change** | | | ***P* value** | | | ***Q**** | |  |
| DUSP1 | 5 | | 1.38 | | | 1.50E-04 | | | 0.08 | | | 1.30 | | | | 1.35E-03 | | | 0.06 | | | 1.29 | | | 3.55E-03 | | | 0.11 | |  |
| FGFBP2 | 4 | | 0.75 | | | 3.50E-04 | | | 0.004 | | | 1.37 | | | | 4.00E-04 | | | 0.02 | | | 1.09 | | | 3.25E-01 | | | 1.00 | |  |
| PPP1R15A | 19 | | 1.27 | | | 1.15E-03 | | | 0.31 | | | 1.19 | | | | 3.61E-02 | | | 0.47 | | | 1.29 | | | 1.75E-03 | | | 0.07 | |  |
| NKG7 | 19 | | 0.78 | | | 1.40E-03 | | | 0.35 | | | 1.27 | | | | 3.80E-03 | | | 0.12 | | | 1.07 | | | 4.42E-01 | | | 1.00 | |  |
| FOS | 14 | | 1.26 | | | 2.90E-03 | | | 0.53 | | | 1.29 | | | | 1.15E-03 | | | 0.05 | | | 1.46 | | | 5.00E-05 | | | 0.00 | |  |
| GPR56 | 16 | | 0.75 | | | 7.50E-03 | | | 0.94 | | | 1.31 | | | | 1.05E-03 | | | 0.047 | | | 1.15 | | | 1.07E-01 | | | 1.00 | |  |
| GLRX5 | 14 | | 0.80 | | | 1.32E-02 | | | 1.00 | | | 1.01 | | | | 9.47E-01 | | | 1.00 | | | 0.84 | | | 9.15E-02 | | | 0.73 | |  |
| SLC31A2 | 9 | | 1.30 | | | 5.13E-02 | | | 1.00 | | | 1.24 | | | | 1.31E-01 | | | 1.00 | | | 1.21 | | | 2.01E-01 | | | 1.00 | |  |
| PTGS2 | 1 | | 1.18 | | | 5.49E-02 | | | 1.00 | | | 1.05 | | | | 5.82E-01 | | | 1.00 | | | 1.04 | | | 7.21E-01 | | | 1.00 | |  |
| GZMB | 14 | | 0.80 | | | 6.99E-02 | | | 1.00 | | | 1.13 | | | | 3.78E-01 | | | 1.00 | | | 1.15 | | | 3.42E-01 | | | 1.00 | |  |
| IL2RB | 22 | | 0.86 | | | 7.25E-02 | | | 1.00 | | | 1.09 | | | | 3.29E-01 | | | 1.00 | | | 1.00 | | | 9.75E-01 | | | 1.00 | |  |
| PRF1 | 10 | | 0.88 | | | 1.01E-01 | | | 1.00 | | | 1.07 | | | | 4.43E-01 | | | 1.00 | | | 1.03 | | | 6.95E-01 | | | 1.00 | |  |
| TAGLN2 | 1 | | 1.15 | | | 1.03E-01 | | | 1.00 | | | 1.11 | | | | 2.28E-01 | | | 1.00 | | | 1.28 | | | 8.40E-03 | | | 0.20 | |  |
| VIM | 10 | | 1.15 | | | 1.10E-01 | | | 1.00 | | | 1.15 | | | | 1.01E-01 | | | 1.00 | | | 1.16 | | | 9.62E-02 | | | 0.74 | |  |
| MYADM | 19 | | 1.14 | | | 1.13E-01 | | | 1.00 | | | 1.25 | | | | 8.35E-03 | | | 0.20 | | | 1.26 | | | 1.14E-02 | | | 0.24 | |  |
| CD97 | 19 | | 1.21 | | | 1.20E-01 | | | 1.00 | | | 1.13 | | | | 2.76E-01 | | | 1.00 | | | 1.18 | | | 1.34E-01 | | | 1.00 | |  |
| TAGAP | 6 | | 1.12 | | | 1.98E-01 | | | 1.00 | | | 1.19 | | | | 8.26E-02 | | | 0.70 | | | 1.17 | | | 1.62E-01 | | | 1.00 | |  |
| MCL1 | 1 | | 1.11 | | | 2.42E-01 | | | 1.00 | | | 1.13 | | | | 1.51E-01 | | | 1.00 | | | 1.12 | | | 1.81E-01 | | | 1.00 | |  |
| GRAMD1A | 19 | | 1.15 | | | 2.60E-01 | | | 1.00 | | | 1.02 | | | | 8.47E-01 | | | 1.00 | | | 1.21 | | | 6.87E-02 | | | 0.64 | |  |
| OBFC2A | 2 | | 1.12 | | | 2.80E-01 | | | 1.00 | | | 1.06 | | | | 5.69E-01 | | | 1.00 | | | 1.07 | | | 5.55E-01 | | | 1.00 | |  |
| GNLY | 2 | | 0.89 | | | 3.00E-01 | | | 1.00 | | | 1.15 | | | | 2.20E-01 | | | 1.00 | | | 1.24 | | | 7.71E-02 | | | 0.68 | |  |
| KCNJ2 | 17 | | 1.07 | | | 3.65E-01 | | | 1.00 | | | 1.09 | | | | 3.22E-01 | | | 1.00 | | | 1.12 | | | 2.03E-01 | | | 1.00 | |  |
| CLC | 19 | 1.08 | | 4.56E-01 | | | 1.00 | | | 1.15 | | | | 2.34E-01 | | | 1.00 | | | 1.16 | | | 2.27E-01 | | | 1.00 | | |  |  |
| S100A10 | 1 | 1.06 | | 4.76E-01 | | | 1.00 | | | 1.21 | | | | 3.91E-02 | | | 0.49 | | | 1.24 | | | 1.73E-02 | | | 0.31 | | |  |  |
| ANXA1 | 9 | 1.06 | | 4.99E-01 | | | 1.00 | | | 1.10 | | | | 3.14E-01 | | | 1.00 | | | 1.14 | | | 3.20E-01 | | | 1.00 | | |  |  |
| ANTXR2 | 4 | 1.09 | | 5.00E-01 | | | 1.00 | | | 1.10 | | | | 5.19E-01 | | | 1.00 | | | 1.22 | | | 2.55E-01 | | | 1.00 | | |  |  |
| AHNAK | 11 | 0.92 | | 5.48E-01 | | | 1.00 | | | 1.11 | | | | 2.24E-01 | | | 1.00 | | | 1.13 | | | 2.22E-01 | | | 1.00 | | |  |  |
| TMEM43 | 3 | 1.07 | | 5.64E-01 | | | 1.00 | | | 1.11 | | | | 3.97E-01 | | | 1.00 | | | 1.17 | | | 2.10E-01 | | | 1.00 | | |  |  |
| TIPARP | 3 | 0.96 | | 6.68E-01 | | | 1.00 | | | 1.14 | | | | 1.89E-01 | | | 1.00 | | | 1.10 | | | 3.65E-01 | | | 1.00 | | |  |  |
| BHLHE40 | 3 | 1.03 | | 7.01E-01 | | | 1.00 | | | 1.13 | | | | 1.48E-01 | | | 1.00 | | | 1.03 | | | 7.41E-01 | | | 1.00 | | |  |  |
| PIGB | 15 | 1.11 | | | 7.65E-01 | | | 1.00 | | | 1.22 | | | | 1.35E-01 | | | 1.00 | | | 1.14 | | | 3.45E-01 | | | 1.00 | | | |
| ARHGAP15 | 2 | 1.10 | | | 8.09E-01 | | | 1.00 | | | 1.05 | | | | 8.59E-01 | | | 1.00 | | | 1.16 | | | 5.85E-01 | | | 1.00 | | | |
| FBXL5 | 4 | 1.00 | | | 9.73E-01 | | | 1.00 | | | 1.02 | | | | 8.53E-01 | | | 1.00 | | | 1.08 | | | 5.12E-01 | | | 1.00 | | | |
| HAVCR2 | 5 | 1.01 | | | 9.74E-01 | | | 1.00 | | | 1.05 | | | | 7.82E-01 | | | 1.00 | | | 0.95 | | | 8.04E-01 | | | 1.00 | | | |

**Table S3**. Differences in baseline expression levels for *FOS, DUSP1* and *PPP1R15A* between thiazide diuretics responders and non-responders in PEAR and PEAR-2 with adjustment for age, gender and baseline blood pressure

|  | **HCTZ Whites** | | **Chlorthalidone Whites** | | **Chlorthalidone Blacks** | |
| --- | --- | --- | --- | --- | --- | --- |
| **Genes** | **Fold Change** | ***P* value** | **Fold Change** | ***P* value** | **Fold Change** | ***P* value** |
| FOS | 1.23 | 0.0334 | 1.23 | 0.0454 | 1.3 | 0.069 |
| DUSP1 | 1.45 | 0.0242 | 1.23 | 0.0466 | 1.18 | 0.14 |
| PPP1R15A | 1.28 | 0.0025 | 1.14 | 0.1632 | 1.2 | 0.071 |

Generalized linear model implemented in edgeR^21^

Fold change corresponds to gene expression levels in responders divided by levels in non-responders, in fragments per kilobase per million reads (FPKM)


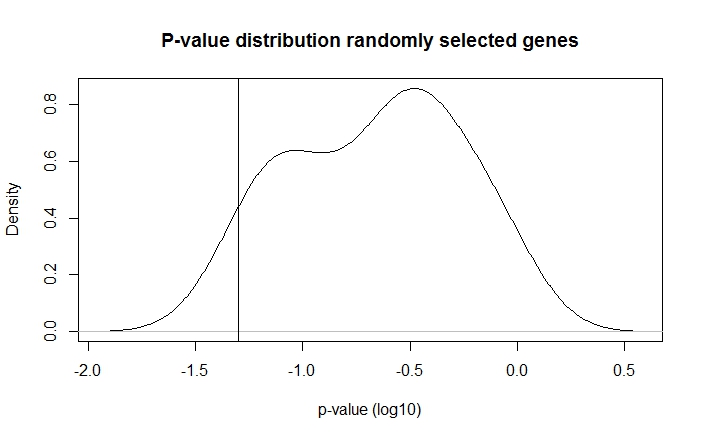


**Figure S2**. P-value distribution for association with thiazide diuretics blood pressure response for 20 randomly selected genes (sets of 3 genes randomly selected from the whole transcriptome). The area to left of the vertical line marks p-values < 0.05 (p-value log_10_ < -1.3). Statistical model including thiazide diuretic (TD) genes *FOS, DUSP1* and *PPP1R15A* showed superior prediction of drug response (P = 0.02) than models with randomly selected gene signature (P range: = 0.045 - 0.96).

**Table S4**. Representative *trans* eQTL for top differentially expressed genes and association with BP response to thiazide diuretics in PEAR whites and PEAR-2 whites and blacks

|  | **SNP - Gene Association*** | | | | **PEAR whites participants** | | | | | | **PEAR2 whites participants** | | | | | | **PEAR2 blacks participants** | | | | | |
| --- | --- | --- | --- | --- | --- | --- | --- | --- | --- | --- | --- | --- | --- | --- | --- | --- | --- | --- | --- | --- | --- | --- |
|  | FOS | | PPP1R15A | | HCTZ DBP | | | HCTZ SBP | | | CLTD DBP | | | CLTD SBP | | | CLTD DBP | | | CLTD SBP | | |
| **SNP** | Z score | P value | Z score | P value | *β* | SE | *P* | *β* | SE | *P* | *β* | SE | *P* | *β* | SE | *P* | *β* | SE | *P* | *β* | SE | *P* |
| rs11065987 | -5.4 | 5.6E-08 | -4.7 | 2.8E-06 | -1.4 | 0.5 | 2.9E-03 | -2.1 | 0.7 | 1.8E-03 | -0.5 | 0.5 | 0.36 | -0.1 | 0.8 | 0.85 | 1.1 | 1.4 | 0.426 | 2.5 | 2.1 | 0.24 |

*Data from Blood eQTL database^22^

SNP, single nucleotide polymorphism; HCTZ, hydrochlorothiazide; CLTD, chlorthalidone; SBP, systolic blood pressure and DBP, diastolic blood pressure


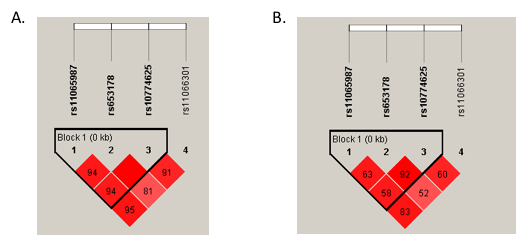


**Figure S3**. Linkage disequilibrium plots between rs10655987, rs653178, rs10774625 and rs11066301 single nucleotide polymorphisms. Linkage disequilibrium is represented in r^2^ (A) and D’(B) values with data from the 1000 Genome project, phase 3 release CEU population using Haploview[^1^](#_ENREF_1).

**References**:

1. Barrett JC, Fry B, Maller J, Daly MJ. Haploview: Analysis and visualization of ld and haplotype maps. *Bioinformatics*. 2005;21:263-265
